# Understanding and Managing Infusion Reactions and Hypophosphataemia With Intravenous Iron—A Nurses' Consensus Paper

**DOI:** 10.1002/nop2.70191

**Published:** 2025-03-26

**Authors:** Aileen Fraser, Vida Cairnes, Else Mikkelsen, Christina Knellwolf, Regula Locher, Marie Andersson

**Affiliations:** ^1^ University Hospitals Bristol NHS Foundation Trust Bristol UK; ^2^ Department of Gastroenterology Royal Devon University Healthcare NHS Foundation Trust Exeter UK; ^3^ Department of Gastroenterology Regional Hospital Gødstrup Herning Denmark; ^4^ Center for Neuromuscular Diseases/ALS Clinic Kantonsspital St. Gallen St. Gallen Switzerland; ^5^ Center for Gastroenterology and Hepatology Zürich Switzerland; ^6^ Department of Gastroenterology Västra Götalandsregionen Borås Sweden

**Keywords:** fatigue, hypersensitivity reactions, hypophosphataemia, intravenous iron, nursing, safety

## Abstract

**Aim:**

To provide evidence‐based guidance on practical aspects and potential safety concerns (infusion reactions and hypophosphataemia) related to the use of intravenous iron from a nursing perspective.

**Design:**

A modified Delphi consensus method.

**Methods:**

Literature searches were conducted and used to support the development of 16 consensus statements. Six nurses with expertise in the field of gastroenterology and experience with the administration of intravenous iron participated in a modified Delphi process to develop a final set of statements.

**Results:**

Overall, 16 statements achieved consensus and covered the practicalities of administration, infusion reactions and hypophosphataemia. Patient preparation is a key step in the administration of intravenous iron, but information should be communicated carefully to prevent undue anxiety. Highlighting the nurse's confidence in the management of any reactions may help to reduce anxiety. The patient should be observed during the first 5–10 min of an infusion to allow prompt management of immediate infusion reactions, although severe hypersensitivity reactions are rare. Nurses should be vigilant for symptoms of hypophosphataemia (such as fatigue, weakness and muscle/bone pain), which can develop following treatment with ferric carboxymaltose, saccharated ferric oxide and iron polymaltose. Serum phosphate levels should be measured in patients receiving ferric carboxymaltose who are at risk of low phosphate.

**Impact:**

Infusion reactions and hypophosphataemia with intravenous iron are documented in the literature, but existing publications do not approach these topics from a nursing perspective. This consensus paper highlights the importance of patient preparation, monitoring and prompt management when administering intravenous iron to ensure patient safety. Considering that nurses have a central role in the administration of intravenous iron, the availability of evidence‐based guidance is essential for both nurse confidence and patient safety.

**Patient or Public Contribution:**

No patient or public contribution was involved in the consensus process.

## 1 Introduction

Anaemia is a major global health issue that is often caused by iron deficiency (ID) (Safiri et al. 2021). Various physiologic, environmental, pathologic and genetic factors can disrupt iron homeostasis and result in ID and/or iron deficiency anaemia (IDA) (Cappellini et al. 2020). ID/IDA is frequently observed in patients with chronic conditions, such as inflammatory bowel disease (IBD), chronic kidney disease or chronic heart failure, but it may also occur postoperatively (such as after bariatric surgery), during pregnancy or following treatment with some drugs (e.g., chemotherapy) (Cappellini et al. 2020; NHLBI 2021). ID/IDA has been associated with a wide range of clinical symptoms. Common signs and symptoms include fatigue, pallor, headache, shortness of breath, pagophagia (a craving for ice) and restless legs syndrome (Lopez et al. 2016; Achebe et al. 2023). Worsening anaemia can lead to more serious consequences, including neurological damage and arrhythmias (NHLBI 2021).

Together with signs and symptoms, a diagnosis of IDA is confirmed by laboratory testing (NHLBI 2021). Though definitions may vary across age groups and according to life stage (e.g., pregnancy) or the presence of chronic conditions, IDA in adults is typically defined as a haemoglobin (Hb) level < 12/13 g/dL (female/male) with a serum (*s*)‐ferritin level < 30 μg/L or an *s*‐ferritin level < 100 μg/L with a transferrin saturation (TSAT) < 20% (KDIGO 2012; Dignass et al. 2015; RCN 2019). Clinical guidelines also highlight the importance of considering inflammation when making a diagnosis; in the presence of inflammation, *s*‐ferritin measurements are an unreliable indicator of IDA (KDIGO 2012; Dignass et al. 2015; RCN 2019). ID in the absence of anaemia is estimated to be twice as common as IDA (Camaschella 2019) and is similarly confirmed by laboratory parameters: Hb ≥ 12/13 g/dL (female/male), with *s*‐ferritin < 30 μg/L or an *s*‐ferritin level < 100 μg/L with a TSAT < 20% (KDIGO 2012; Dignass et al. 2015).

## 2 Background

For uncomplicated IDA, oral iron can be considered as a first‐line treatment (RCN 2019). However, intravenous (IV) iron is the first‐line treatment where oral iron is ineffective or cannot be used, or where there is a need for rapid iron replacement: for example, in patients with active IBD; previous intolerance to oral iron; an Hb level < 10 g/dL or in those receiving erythropoiesis‐stimulating agents (Dignass et al. 2015; RCN 2019). When IV iron is indicated, dosing may be informed by recommendations in the product information and clinical judgement.

Although modern IV iron formulations are widely used for the treatment of IDA across various therapeutic indications, some clinicians remain wary of using IV iron due to negative perceptions of its safety (Auerbach and Deloughery 2016; Blumenstein et al. 2021). These safety concerns primarily relate to the potential for severe hypersensitivity reactions (HSRs) (inappropriate or exaggerated immune responses) (Justiz Vaillant et al. 2022) with IV iron, which were more common with high molecular weight iron dextran (HMWID) – a formulation that was withdrawn from the market more than a decade ago (Auerbach and Deloughery 2016; Blumenstein et al. 2021). Evidence suggests that the attempt to prevent infusion‐related reactions by premedicating with corticosteroids or antihistamines may induce symptoms mimicking a mild HSR, potentially increasing the frequency of adverse events reported with IV iron (Arastu et al. 2022). With modern IV iron formulations, the risk of severe HSRs is very low (Chertow et al. 2006; Avni et al. 2015) and no differences between formulations have been detected (Achebe and DeLoughery 2020). An additional concern is the potential for hypophosphataemia (serum phosphate < 2.0 g/dL) (Blumenstein et al. 2021), but symptomatic hypophosphataemia is primarily associated with specific IV iron formulations (Zoller et al. 2017; Schaefer, Tobiasch, et al. 2022).

While HSRs and hypophosphataemia have been extensively reported in the literature, existing publications do not approach these topics from a nursing perspective. In clinical practice, mild/moderate and severe infusion reactions are not always correctly distinguished, leading to inappropriate event management (Achebe and DeLoughery 2020). Furthermore, hypophosphataemia often goes unnoticed due to a lack of awareness of this side effect, with action taken only when serious consequences have already occurred. In addition, advice regarding patient preparation is particularly limited, despite the importance of these aspects to nurses who administer IV iron.

## 3 The Study

### 3.1 Aim and Objective

To address these gaps in the literature, a consensus group of nurses was convened to provide evidence‐based information and guidance regarding practical aspects and safety concerns related to IV iron administration.

## 4 Methods

### 4.1 Modified Delphi Consensus Process

A consensus group was established, which comprised six nurses with expertise in the field of gastroenterology and experience with administering and/or prescribing IV iron. A modified Delphi process was initiated at a face‐to‐face meeting in Copenhagen, Denmark, on 9 October 2022. During this meeting, potential topics for inclusion in the consensus paper were discussed.

A set of 16 statements covering practical aspects of IV iron administration, and infusion reactions and hypophosphataemia with IV iron treatment were drafted. Literature searches of the PubMed/Medline database were conducted to support the development of the consensus statements by identifying publications addressing safety issues (infusion reactions or hypophosphataemia) with IV iron. Search terms comprised: (((FCM OR ‘Ferric carboxymaltose’) OR (IIM OR ‘Ferric derisomaltose’) OR (‘iron sucrose’) OR (‘iron dextran’) OR (‘ferumoxytol”) OR (‘sodium ferric gluconate’) OR (SFO OR ‘saccharated ferric oxide’) OR (IPM OR ‘iron polymaltose’)) AND (‘iron‐deficient’[title/abstract] OR ‘iron deficiency’ [title/abstract])) AND (*sensitiv*[tiab] OR hypophos*[tiab]). Outputs of the searches were limited to the previous 10 years and were screened for relevance.

To determine the extent of the group's agreement with the draft statements, a survey was built using SurveyMonkey (Momentive Inc., San Mateo, CA). In the survey, participants were asked to indicate their level of agreement with each statement on a 9‐point Likert scale in line with the consensus process reported in (Rosenfeld et al. 2015). The 9‐point scale included the following anchors: 1 = strongly disagree; 3 = disagree; 5 = neutral; 7 = agree and 9 = strongly agree. A comment box was also provided with each statement to allow participants to provide suggestions for changes.

The survey was distributed to the consensus group on 20 January 2023 together with a document reminding the nurses of the consensus process. After a 12‐day response period, the results were analysed. Findings from the survey were discussed at a follow‐up meeting in London, UK, on 4 February 2023. Consensus was defined as a mean score of ≥ 7.0 with ≤ 1 outlier (a rating ≥ 2.0 points from the mean) and agreement was defined as the proportion of ratings ≥ 7.0 points. Due to the high level of consensus among the group, a second consensus survey was not considered necessary; the nurses reviewed and approved the final statements in the consensus paper.

## 5 Results and Discussion

Based on the responses to the survey, all statements achieved consensus. A total of 12 statements achieved 100% agreement, 2 statements (5.1.2 and 5.1.4) achieved 83.3% agreement and 2 statements (5.3.1 and 5.3.3) achieved 80% agreement. The final statements included in this consensus paper were approved by all nurses.

### 5.1 Practical Aspects of IV Iron Administration

Statements in the following section address practical aspects of IV iron administration that are particularly relevant for nurses. Due to the lack of literature providing specific guidance for nurses, the statements are primarily based on expert consensus.

#### 5.1.1 When Preparing a Patient for an Intravenous Iron Infusion, Establishing Trust and an Open Dialogue With the Patient Are Important

Patient education is a primary step in the administration of an IV iron infusion (Lim et al. 2019), and is necessary to obtain informed consent and gain patient trust. Typically, this task is undertaken by the prescriber prior to the infusion. On the day of the infusion, the nurse can focus on managing the patient's expectations, ensuring that all relevant information has been received and understood, and that the patient knows the purpose of the treatment, its risks and its benefits.

#### 5.1.2 Before Administering an Intravenous Iron Infusion, It Is Important That the Patient Understands That Immediate Side Effects and Reactions Are Not Expected During the Infusion; but, if They Do Occur, the Nurse Knows How to Manage Them

As part of the education process prior to the infusion, the patient should be made aware of the possibility of infusion reactions (Lim et al. 2019); however, this should be communicated carefully to avoid undue anxiety. Psychological burden, such as anxiety of the patient, has been associated with an increased risk of severe reactions to IV infusions (Worm et al. 2018; Lim et al. 2019). Reassuring the patient that they will be monitored (Lim et al. 2019) and highlighting the nurse's confidence and competence in the management of any reactions may help to reduce anxiety.

#### 5.1.3 Before Administering an Intravenous Iron Infusion, the Patient Should Know Who to Contact if They Feel Unwell in the Hours, Days or Weeks After Administration

It is important that the patient knows who to contact if they feel unwell following an IV iron infusion; typically, this will be their local outpatient clinic or general practitioner. While delayed reactions to IV iron are not well documented, they have been known to occur a few hours or days after an IV iron infusion (Achebe and DeLoughery 2020). Such reactions may manifest as mild flu‐like symptoms characterised by joint pain, muscle aches and sometimes fever (Achebe and DeLoughery 2020). In addition, symptoms of hypophosphataemia (e.g., fatigue and weakness) (Zoller et al. 2017) may arise in the days or weeks following the administration of certain IV iron formulations.

#### 5.1.4 It Is Important to Establish Local Practice Guidelines for the Administration of Intravenous Iron and to Regularly Review and Update Guidelines in Line With Current Evidence

If available, local practice guidelines for the administration of IV iron and the management of infusion reactions should be followed. Where local protocols are unavailable, it is important that they are introduced and refreshed at regular intervals in line with existing literature. Routine audits can be useful to ensure that existing protocols reflect the current evidence, are effective and are being followed.

### 5.2 Infusion Reactions With IV Iron

Infusion reactions can occur during or shortly after the administration of IV iron (Achebe and DeLoughery 2020). The aetiology of infusion reactions can vary. In some cases, reactions can be due to hypersensitivity (i.e., an HSR), which is defined as an inappropriate or exaggerated immune response to an antigen or allergen (Justiz Vaillant et al. 2022). In the context of IV iron administration, infusion reactions are graded based on their clinical presentation (Rampton et al. 2014). Mild reactions (e.g., Fishbane reactions) are associated with symptoms such as flushing, chest pain and back pain (Rampton et al. 2014; Achebe and DeLoughery 2020). Symptoms of a moderate reaction include those of a mild reaction with more pronounced chest tightness and shortness of breath; additionally, changes in vital signs may be observed (e.g., increased heart rate and decreased blood pressure) (Rampton et al. 2014). Severe reactions refer to anaphylactic reactions, which are generalised or systemic HSRs that rarely occur with IV iron infusion (Rampton et al. 2014; Achebe and DeLoughery 2020; Cardona et al. 2020).

#### 5.2.1 The Incidence of Moderate‐to‐Severe Hypersensitivity Reactions With Intravenous Iron Is Low

Some clinicians have concerns regarding the risk of HSRs with IV iron (Auerbach and Deloughery 2016). However, these concerns are based on the historical use of HMWID, which was frequently associated with severe infusion reactions (Auerbach and Deloughery 2016). Serious or severe HSRs with modern IV iron formulations are rare (Chertow et al. 2006; Rampton et al. 2014; Avni et al. 2015; Wolf et al. 2021).

A pooled analysis was conducted to identify differences in the incidence of moderate‐to‐severe HSRs between ferric carboxymaltose (FCM), ferric derisomaltose (FDI), ferumoxytol and iron sucrose (IS) – the four most widely used IV iron formulations in Europe and the US (Achebe and DeLoughery 2020). The analysis included five head‐to‐head randomised controlled trials (RCTs) that were powered to evaluate HSRs as pre‐specified primary or secondary endpoints (Achebe and DeLoughery 2020). Overall, the incidence of moderate‐to‐severe HSRs was low (0.2%–1.7%), and no significant risk differences were identified between the formulations (Achebe and DeLoughery 2020). Similarly, in a meta‐analysis of data from 103 RCTs, no IV iron formulation (except ferric gluconate) was associated with a significantly increased risk of severe infusion reactions relative to comparators (i.e., no iron, placebo, oral iron or intramuscular iron) (Avni et al. 2015). In a more recent meta‐analysis of RCTs that investigated the incidence of HSRs using standardised Medical Dictionary for Regulatory Activities terms, serious or severe HSRs were uncommon with FDI (0.14%) and FCM (1.08%) (Kennedy et al. 2023). Findings from RCTs are supported by real‐world experience; in a large, retrospective, observational study that included 7354 patients who received IV iron (FDI), the incidence of clinician‐validated severe HSRs was < 0.1% (Sinclair et al. 2022).

#### 5.2.2 Mild/Moderate and Severe Reactions With Intravenous Iron Are Distinguished by Monitoring Signs and Symptoms and Their Speed of Development

Evaluating the signs and symptoms of a reaction to IV iron, and monitoring their speed of development, can help to distinguish mild/moderate reactions from severe reactions (Table 1).

**TABLE 1:** Signs, symptoms and the speed of development of mild/moderate and severe infusion reactions.

	Mild/moderate reactions	Severe reactions
Reaction	Mild (e.g., Fishbane) or moderate reactions	Severe hypersensitivity
Signs and symptoms	Fishbane—flushing, back or chest pain, chest tightness, sometimes with shortness of breath Isolated signs and symptoms—hives, itching, rash, mild low or high blood pressure, increased heart rate, nausea or headache	Persistent low blood pressure (a drop of 30 mmHg SBP from baseline, or SBP < 90 mmHg), OR swelling of the tongue/airway, OR two or more cardiovascular (e.g., chest pain), skin (e.g., generalised hives), respiratory (e.g., noisy breathing) and/or gastrointestinal (e.g., vomiting) symptoms
Speed of development	Typically develop during the first few minutes of an infusion. Moderate reactions may develop from milder reactions	May develop suddenly or following the progressive worsening of mild/moderate symptoms
Outcome after the infusion is stopped	Symptoms are self‐limiting and most abate soon after the infusion is stopped. Isolated symptoms, such as hives, may need treatment with antihistamine to resolve	Symptoms typically persist or continue to worsen without intervention

*Note:* Table developed based on information from (Rampton et al. 2014; Lim et al. 2019; Achebe and DeLoughery 2020; Cardona et al. 2020).

Abbreviation: SBP, systolic blood pressure.

#### 5.2.3 If a Reaction Occurs With Intravenous Iron, It Is Appropriate to Implement a Management Approach and/or Treatment Plan According to the Type of Reaction

**FIGURE 1:**
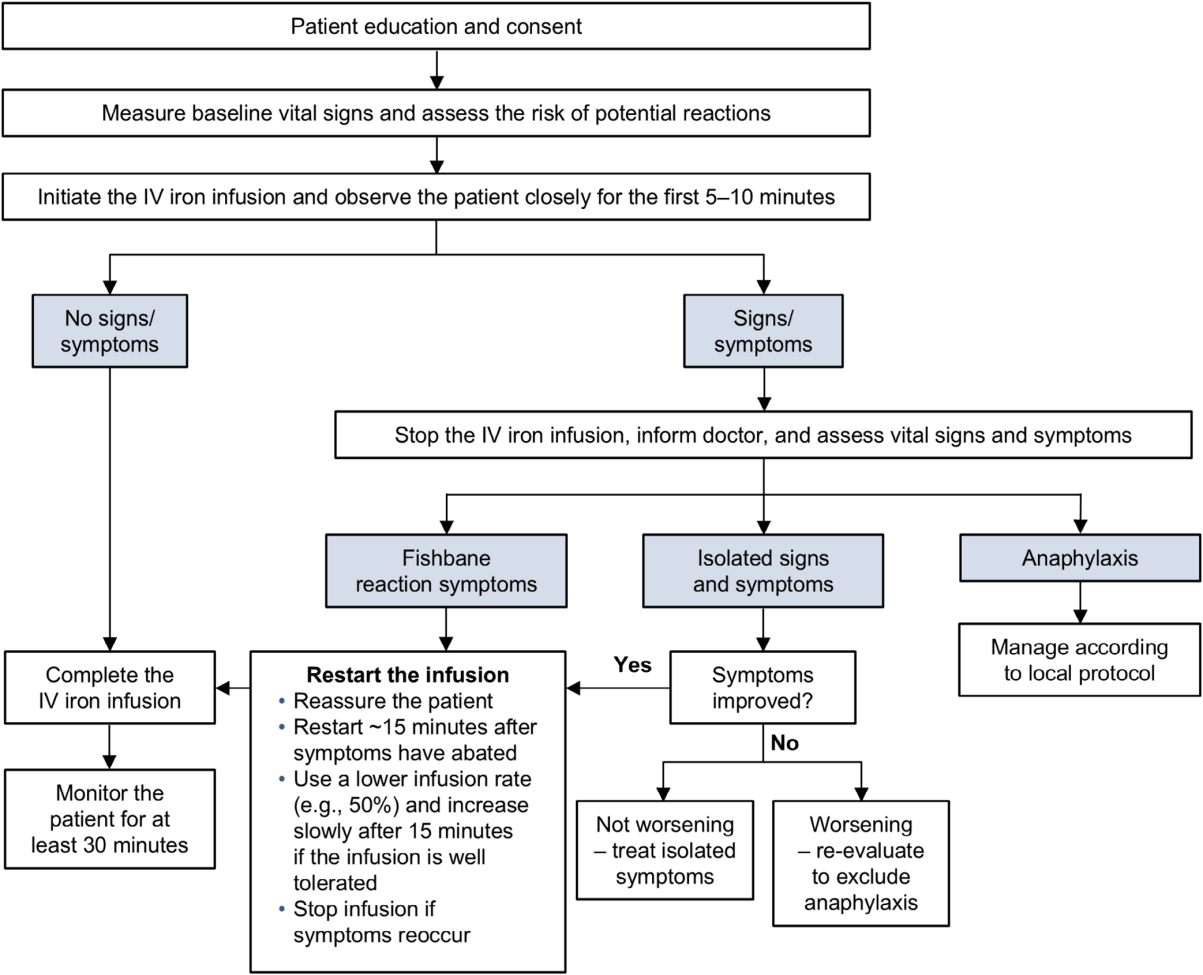
Algorithm for the administration of IV iron and the management of potential infusion reactions. Adapted from Achebe M, DeLoughery TG. 2020. *Transfusion*. 60, no. 6: 1154–1159. https://doi.org/10.1111/trf.15837, licensed under CC‐BY‐NC (https://creativecommons.org/licenses/by‐nc/4.0/), which permits use, distribution and reproduction in any medium, provided the original work is properly cited and is not used for commercial purposes. IV = intravenous.

A simple algorithm providing evidence‐based guidance regarding the management of infusion reactions is presented in Figure 1. With any reaction, timely diagnosis and management are crucial. However, it is also important to avoid reactionary interventions such as administering drugs before assessing all symptoms and vital signs; inappropriate intervention has the potential to escalate mild infusion reactions (Auerbach and Deloughery 2016).

#### 5.2.4 Fishbane Reactions (Mild Reactions) Will Abate After the Intravenous Iron Infusion Is Stopped; the Infusion Can Be Restarted at a Lower Rate Once the Symptoms Have Subsided

The symptoms of Fishbane reactions abate after the IV iron infusion is stopped and typically do not reappear when the infusion is restarted (Rampton et al. 2014; Achebe and DeLoughery 2020). After providing reassurance and receiving patient consent, the infusion can be restarted at a lower rate after the symptoms have disappeared (Figure 1) (Achebe and DeLoughery 2020).

#### 5.2.5 Certain Comorbidities (e.g., Severe Asthma, Eczema or Other Atopic Allergies) or a Previous Reaction to an Intravenous Iron Formulation May Increase the Likelihood of Experiencing a Hypersensitivity Reaction

Risk factors for HSRs with IV iron include a history of severe asthma, eczema or other atopic diseases, mastocytosis, and systemic inflammatory diseases (Rampton et al. 2014). Other factors, such as severe respiratory or cardiac disease and the use of beta‐blockers or angiotensin‐converting enzyme inhibitors, can increase the severity of an HSR if it does occur (Lim et al. 2019; Rampton et al. 2014).

Patients with a specific contraindication to IV iron are also at higher risk of experiencing HSRs; such contraindications include known hypersensitivity to the active substance or excipients of the IV iron formulation (European Medicines Agency (EMA) 2013; Rampton et al. 2014). This emphasises the importance of prescribing and administering IV iron in line with the product information.

#### 5.2.6 Although Hypersensitivity Reactions Cannot Be Predicted or Completely Prevented, the Risk May Be Minimised Through Careful Risk–Benefit Assessment in Patients More Likely to Experience a Hypersensitivity Reaction (e.g., in Those With a History of Atopic Disease) and by Careful Monitoring of All Patients During an Infusion

To ensure that an appropriate risk–benefit assessment is made, patients with an increased risk of experiencing an HSR or a more severe HSR can be identified prior to infusion (Rampton et al. 2014). In some cases, additional vigilance during the infusion is prudent and a slower infusion rate may be necessary (Rampton et al. 2014). The EMA also recommends that IV iron is administered only when resuscitation facilities and staff trained to assess and manage HSRs are immediately available (EMA 2013). Observing patients during the first 5–10 min of an infusion enables appropriate and timely management of immediate reactions (Van Doren et al. 2024). Additionally, detailed documentation of any HSR can inform the future treatment strategy for the patient and is important for pharmacovigilance (Rampton et al. 2014; Lim et al. 2019).

### 5.3 Hypophosphataemia

Hypophosphataemia is increasingly recognised as a potential clinically relevant side effect of certain IV iron formulations (Glaspy et al. 2021). Clinically, hypophosphataemia is defined as a serum phosphate level < 2.5 mg/dL (< 0.81 mmol/L): 2.0–< 2.5 mg/dL (0.65–< 0.81 mmol/L) for mild phosphate deficiency, 1–< 2.0 mg/dL (0.32–< 0.65 mmol/L) for moderate and < 1 mg/dL (< 0.32 mmol/L) for severe (Rosano et al. 2020).

#### 5.3.1 The Incidence of Hypophosphataemia Is Generally Low With Intravenous Iron Formulations, Except for Ferric Carboxymaltose, Iron Polymaltose and Saccharated Ferric Oxide, for Which High Incidence Rates Have Been Reported

A large and growing number of RCTs, observational studies and case reports have identified hypophosphataemia as a potential side effect of certain IV iron treatments (Schouten et al. 2009; Wolf et al. 2013, 2018, 2020; Zoller et al. 2017; Zoller, Wagner, et al. 2023; Detlie et al. 2019; Emrich et al. 2020; Glaspy et al. 2020; Schaefer et al. 2021; Schaefer, Tobiasch, et al. 2022; Vilaca et al. 2022). The evidence shows that the risk of hypophosphataemia clearly varies according to the formulation administered (Schaefer et al. 2016; Schaefer, Zoller, et al. 2022; Zoller et al. 2017). According to data from prospective head‐to‐head trials, the reported incidence of hypophosphataemia is high with FCM (up to 75.0%) and saccharated ferric oxide (SFO) (up to 83.2%), while low rates are reported for FDI (up to 8.4%), ferumoxytol (up to 0.9%), iron dextran (0.0%) and IS (up to 2.3%), varying across different patient populations (Wolf et al. 2013, 2018, 2020; Adkinson et al. 2018; Auerbach et al. 2019; Emrich et al. 2020; Bhandari et al. 2021; Kawabata et al. 2022; Zoller, Wolf, et al. 2023).

#### 5.3.2 It Is Important to Be Aware of the Symptoms and Complications of Hypophosphataemia That May Occur Following Administration of Certain Intravenous Iron Preparations. Symptoms and Complications Reported in Multiple Case Studies Include Fatigue, Weakness, Muscle Pain, Bone Pain, Osteomalacia and Fractures

**FIGURE 2:**
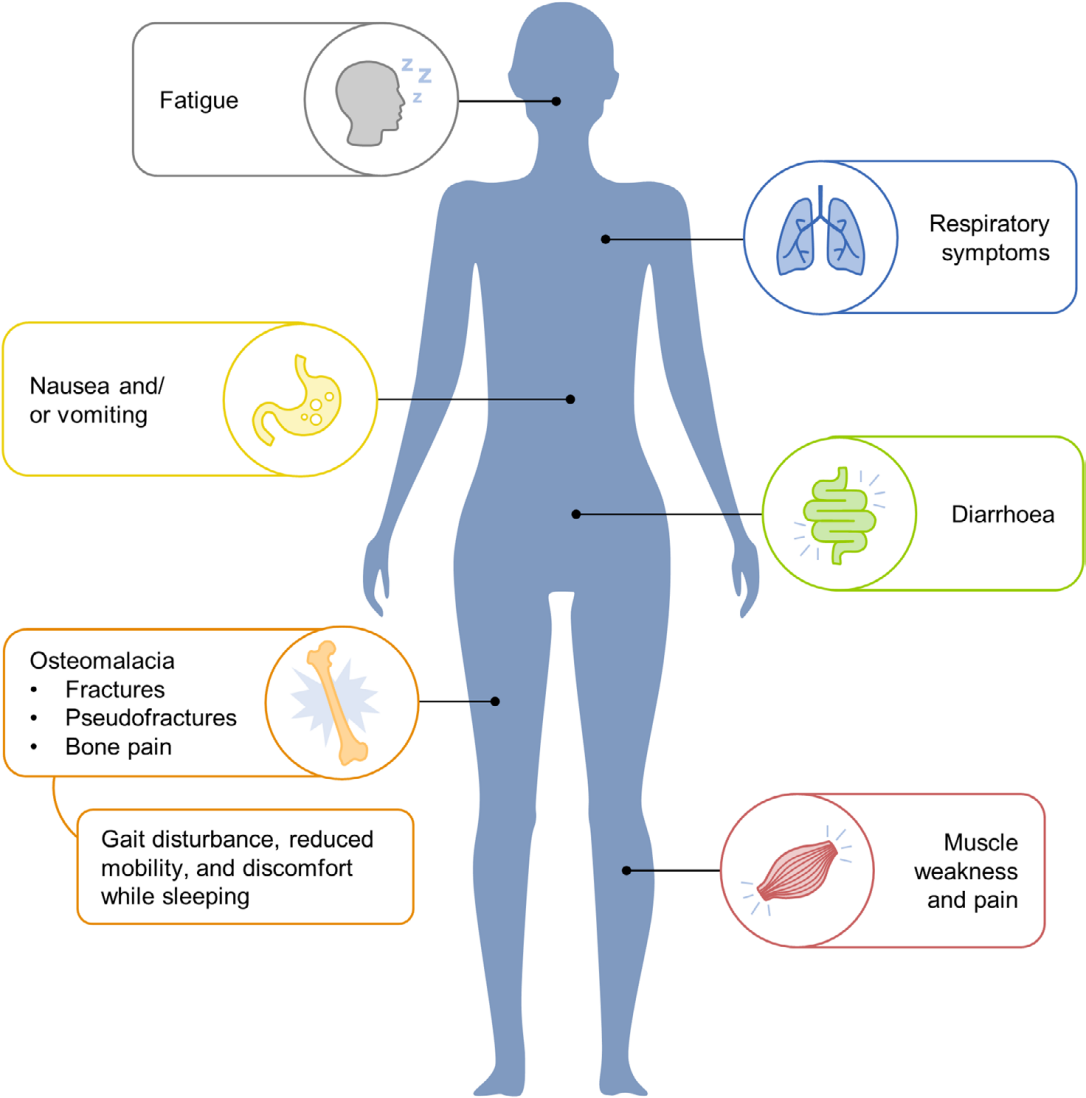
Symptoms and complications of hypophosphataemia following IV iron treatment. Figure developed based on Bishay et al. (2017); Zoller et al. (2017); Bartko et al. (2018); Burckhardt (2018); Klein et al. (2018); Tournis et al. (2018); Urbina et al. (2018); Fang et al. (2019); Callejas‐Moraga et al. (2020); Madero Velázquez et al. (2022); Schaefer, Tobiasch, et al. (2022); Vilaca et al. (2022); Strubbe et al. (2025); von Brackel et al. (2025).

A variety of short‐ and long‐term symptoms and complications have been associated with hypophosphataemia (Figure 2) (Zoller et al. 2017; Schaefer, Tobiasch, et al. 2022; Vilaca et al. 2022; von Brackel et al. 2025).

#### 5.3.3 Hypophosphataemia Following Treatment With Intravenous Iron Is Transient With Most Intravenous Iron Formulations but Has Been Reported to Persist for Weeks or Months Following a Single Dose of Ferric Carboxymaltose

After a single infusion of IV iron, hypophosphataemia is usually transient and asymptomatic (Zoller et al. 2017; Strubbe et al. 2025). However, although initial research suggested that hypophosphataemia following FCM treatment typically lasted 2–3 weeks, more recent research has shown that it can persist for a substantially longer time period, particularly if treatment is repeated (Hardy and Vandemergel 2015; Glaspy et al. 2021; Schaefer, Tobiasch, et al. 2022; Schaefer, Zoller, et al. 2022).

In the PHOSPHARE‐IDA trials, the incidence of hypophosphataemia was 8.0% with FDI and 74.4% with FCM (Wolf et al. 2020; Schaefer, Zoller, et al. 2022). At Day 35, 40.0% of all FCM‐treated patients had persistent hypophosphataemia (Wolf et al. 2020; Schaefer, Zoller, et al. 2022). The prospective observational Oslo study, which included ~100 patients who received a single dose of FCM or FDI, reported the persistence of hypophosphataemia at Week 6 in 21.6% of patients treated with FCM (Detlie et al. 2019). In a retrospective study of 162 patients who received FCM in UK clinical practice, the incidence of moderate/severe hypophosphataemia post‐infusion was 33.7%, which persisted in ~35% of patients at the 61‐ to 90‐day time point (Fragkos et al. 2020). Furthermore, in a retrospective review of the medical records of 130 patients treated with FCM or IS, the duration of hypophosphataemia was 2–18 weeks with ISF and 2–9 months with FCM (Hardy and Vandemergel 2015). The investigators hypothesised that the long duration of hypophosphataemia following FCM treatment may be due to the frequent repetition of treatment during follow‐up (Hardy and Vandemergel 2015). The authors added that some patients who received FCM did not achieve a normal phosphate measurement at any time during the 2‐year study period (Hardy and Vandemergel 2015).

#### 5.3.4 Fatigue Is a Symptom of Iron Deficiency and Can Also Be a Symptom of Hypophosphataemia. Although It Can Be Difficult to Measure Fatigue in the Clinic, Simple Tools Can Facilitate Assessments and May Be Useful When Monitoring Patients With Iron Deficiency and/or Hypophosphataemia

Fatigue is a symptom that can have a variety of potential causes including, but not limited to, depression, anxiety and sleep disorders (Maisel et al. 2021) as well as ID/IDA and hypophosphataemia (Schaefer, Tobiasch, et al. 2022; Zoller, Wagner, et al. 2023). Fatigue is difficult to assess in the clinic (Maisel et al. 2021), though some assessment tools may be helpful (Table 2).

**TABLE 2:** Tools for the assessment of fatigue in clinical practice.

Tool	Details	References
FSS	Self‐assessment questionnaire with nine items evaluating the severity of fatigue in different situations during the past week Validated for the assessment and quantification of fatigue in healthy individuals and in patients with various disorders (MS, ischaemic stroke and sleep–wake disorders)	Valko et al. (2008)
FACIT‐Fatigue	Self‐assessment questionnaire with 13 items evaluating the severity and impact of fatigue during the past 7 days Has demonstrated validity in the assessment of fatigue in patients with IDA (Acaster et al. 2015) and has been used to measure fatigue in clinical trials of IV iron (Strauss and Auerbach 2018; Auerbach et al. 2019; Zoller, Wagner, et al. 2023)	Yellen et al. (1997)
IBD‐F	Validated for the assessment of fatigue in patients with IBD Self‐assessment questionnaire with threesections: frequency and severity of fatigue (5 items), experience and impact of fatigue (30 items), and additional issues related tofatigue (free text)	Czuber‐Dochan et al. (2014)

Abbreviations: FACIT‐Fatigue, Functional Assessment of Chronic Illness Therapy – Fatigue; FSS, Fatigue Severity Scale; IBD, inflammatory bowel disease; IBD‐F, IBD‐Fatigue; IDA, iron deficiency anaemia; IV, intravenous; MS, multiple sclerosis.

A high degree of clinical suspicion is warranted when ongoing fatigue, or smaller than expected improvements in fatigue, are reported by patients who have received IV iron for ID/IDA and particularly by those who have received formulations associated with a high risk of hypophosphataemia (e.g., FCM and SFO). In such patients, while persistent fatigue could be attributed to unresolved ID/IDA or underlying chronic disease (e.g., IBD), it has been specifically associated with hypophosphataemia (Zoller, Wolf, et al. 2023). Furthermore, ongoing fatigue may be less apparent to a patient after IV iron treatment, due to iron replenishment improving fatigue overall (Zoller, Wagner, et al. 2023). In the PHOSPHARE‐IBD trial—the first RCT to directly compare the effect of identical doses of FCM and FDI on hypophosphataemia—FCM was associated with a significantly higher incidence of hypophosphataemia than FDI (51.0% vs. 8.3%; *p* < 0.0001) (Zoller, Wolf, et al. 2023). The same trial showed that fatigue improved faster and to a greater extent with FDI than FCM, and that more severe hypophosphataemia was associated with slower improvement in fatigue (Zoller, Wagner, et al. 2023). From a nursing perspective, it is important to ask the patient about their sleep (Maisel et al. 2021) to determine whether fatigue is ongoing despite good quality sleep. Additional laboratory tests (e.g., *s*‐ferritin measurement) may also be warranted if the findings from questioning and physical examination indicate a specific cause (Maisel et al. 2021).

#### 5.3.5 Risk Factors for Developing Hypophosphataemia Following Treatment With Intravenous Iron Include Choice of Intravenous Iron Formulation, Low Phosphate Level Before Infusion, and Various Concomitant Conditions or Medications That Affect Phosphate Homeostasis

Risk factors for developing hypophosphataemia following IV iron infusion are presented in Table 3. Although hypophosphataemia following treatment with IV iron cannot be predicted, the choice of IV iron formulation is a key factor affecting the risk of hypophosphataemia, as outlined in Statement 5.3.1 (Wolf et al. 2018; Fang et al. 2019). In a secondary analysis of data from the PHOSPHARE trials, treatment with FCM was the most significant risk factor for incident hypophosphataemia (Schaefer, Zoller, et al. 2022) and repeated dosing can further increase the risk (Boots and Quax 2022; Strubbe et al. 2025). Consequently, administering multiple infusions of FCM should be carefully considered; hypophosphataemia following the first infusion could be exacerbated if a second infusion is administered (Rosano et al. 2020).

**TABLE 3:** Risk factors for hypophosphataemia following IV iron treatment.

Risk factor	References
Type of IV iron formulation (e.g., FCM, IPM and SFO)	Schouten et al. (2009), Wolf et al. (2018), Fang et al. (2019), Rosano et al. (2020), Boots and Quax (2022), Kawabata et al. (2022), Schaefer, Zoller, et al. 2022, Chu et al. (2023), Zoller, Wagner, et al. (2023)
Number of doses of IV iron (i.e., > 1 infusion)	Fang et al. (2019), Rosano et al. (2020), Boots and Quax (2022), Rosano et al. (2023)
Low phosphate level before each infusion	Schaefer et al. (2016), Wolf et al. (2018), Boots and Quax (2022), Chu et al. (2023)
Low *s‐*ferritin/Hb level before infusion	Rosano et al. (2020), Boots and Quax (2022), Chu et al. (2023), Rosano et al. (2023)
Low body weight	Wolf et al. (2018), Rosano et al. (2020), Boots and Quax (2022) Schaefer, Zoller, et al. 2022
Preserved kidney function	Wolf et al. (2018), Schaefer et al. (2021)
Concomitant conditions that affect phosphate homeostasis (e.g., bariatric surgery, calcium malabsorption, vitamin D deficiency, hyperparathyroidism and solid or haematological tumours)	Fang et al. (2019), Adhikari et al. (2021), Boots and Quax (2022), Schaefer, Tobiasch, et al. (2022)
Use of medications that predispose to hypophosphataemia (e.g., bisphosphonates, chemotherapy and cancer immunotherapy)	Adhikari et al. (2021), Boots and Quax (2022)

Abbreviations: FCM, ferric carboxymaltose; Hb, haemoglobin; IPM, iron polymaltose; IV, intravenous; *s*‐ferritin, serum ferritin; SFO, saccharated ferric oxide.

#### 5.3.6 It Is Important to Measure Phosphate Levels in Patients Receiving Ferric Carboxymaltose Who Are at Risk of Low Phosphate; Checking Phosphate Levels Before an Infusion May Identify Patients at Risk of Developing Hypophosphataemia, and Measurements After Infusion May Help to Prevent the Possible Consequences of Hypophosphataemia

The product labels for FCM recommend monitoring serum phosphate levels in patients at risk for low phosphate and in those who receive multiple doses of FCM or long‐term treatment (Ferinject SPC 2023; Injectafer PI 2023). Similarly, due to reports of symptomatic patient cases, the Medicines and Healthcare products Regulatory Agency (MHRA) has suggested that patients with risk factors for hypophosphataemia and those receiving multiple higher doses of, or long‐term treatment with, FCM should be monitored (MHRA 2020) – a view that is cited in recent guidelines from the European Crohn's and Colitis Organisation and supported by experts (Schaefer, Tobiasch, et al. 2022; Gordon et al. 2023) To determine and manage the risk of hypophosphataemia, it may be prudent to measure phosphate levels prior to and following treatment (Boots and Quax 2022). For IV iron formulations other than FCM, serum phosphate monitoring is not mentioned in the product labels (INFeD PI 2024; Maltofer PI 2021; Venofer SPC 2023; Feraheme PI 2022; Ferrlecit PI 2022; Monofer SPC 2022; Monoferric PI 2022; Venofer PI 2022; Cosmofer SPC 2023; Ferric Derisomaltose 2023 UK SPC). For patients at risk of hypophosphataemia, a formulation with a low risk of hypophosphataemia should be considered.

### 5.4 Strengths and Limitations

The main strengths of this consensus study are its novelty and utility. While safety issues with IV iron have been discussed in the literature, there is a lack of publications exploring these topics from a practical, nursing perspective. Moreover, the consensus statements were developed by a group of nurses with experience administering and/or prescribing IV iron, resulting in practical statements that can be applied in clinical practice. This study is limited by the use of a modified Delphi consensus process, which may be considered more simplistic than the original method. However, it should be noted that the modification of the process primarily stemmed from the high level of consensus among the nurse group. In addition, the relatively small number of nurses included in the panel and their specific expertise in gastroenterology may mean that additional insights on the safety of IV iron in other patient populations may not have been considered. Nonetheless, the general safety considerations around the practicalities of administering IV iron shared from these experienced nurses can be considered widely applicable to all patients.

## 6 Conclusion

Nurses frequently play a central role in the administration, and sometimes in the prescription, of IV iron for the treatment of IDA. As such, knowledge of key safety issues (infusion reactions and hypophosphataemia) that may arise is essential. Although severe HSRs to IV iron are rare, an understanding of how to identify and manage infusion reactions of all severities may improve nurse and patient confidence as well as facilitate favourable treatment outcomes. Patient preparation is essential and ensures that patients can recognise the symptoms of an infusion reaction and understand that it is often possible to restart the infusion if symptoms abate. Observing the patient during the first 5–10 min of the infusion is an important aspect of the nurse's role. There is increasing awareness of the potential for clinically relevant hypophosphataemia following treatment with FCM, iron polymaltose and SFO. As persistent, symptomatic hypophosphataemia can have important clinical implications, nurses are encouraged to remain vigilant when assessing patients who have received IV iron.

## Author Contributions

Conceptualisation: A.F., V.C., E.M., C.K., R.L. and M.A. Writing – review and editing: A.F., V.C., E.M., C.K., R.L. and M.A.

## Ethics Statement

The authors have nothing to report.

## Consent

The authors have nothing to report.

## Conflicts of Interest

A.F. has been a consultant and speaker for Abbvie Ltd. Dr Falk Pharma, Galapagos, Gilead, Janssen, Pfizer, Pharmacosmos, Takeda UK Ltd. and Tillotts. V.C. has been a consultant and speaker for Falk, Ferring, Janssen, Pharmacosmos and Takeda. C.K. has been a consultant and speaker for Abbvie, Pharmacosmos, Takeda and Vifor Pharma. M.A. has been a consultant and speaker for Abbvie, Janssen, Pharmacosmos, Takeda and Tillotts. E.M. and R.L. declare no conflicts of interest.

## Data Availability

Data sharing not applicable to this article as no datasets were generated or analysed during the current study.
